# Anchor Node Localization for Wireless Sensor Networks Using Video and Compass Information Fusion

**DOI:** 10.3390/s140304211

**Published:** 2014-03-03

**Authors:** Dan Pescaru, Daniel-Ioan Curiac

**Affiliations:** 1 Computer and Software Engineering Department, Politehnica University of Timisoara, V. Parvan no. 2, Timisoara 300223, Romania; E-Mail: dan.pescaru@cs.upt.ro; 2 Automation and Applied Informatics Department, Politehnica University of Timisoara, V. Parvan no. 2, Timisoara 300223, Romania

**Keywords:** wireless sensor network, anchor node, localization, magnetometer, video camera, triangulation

## Abstract

Distributed sensing, computing and communication capabilities of wireless sensor networks require, in most situations, an efficient node localization procedure. In the case of random deployments in harsh or hostile environments, a general localization process within global coordinates is based on a set of anchor nodes able to determine their own position using GPS receivers. In this paper we propose another anchor node localization technique that can be used when GPS devices cannot accomplish their mission or are considered to be too expensive. This novel technique is based on the fusion of video and compass data acquired by the anchor nodes and is especially suitable for video- or multimedia-based wireless sensor networks. For these types of wireless networks the presence of video cameras is intrinsic, while the presence of digital compasses is also required for identifying the cameras' orientations.

## 1. Introduction

A wireless sensor network (WSN) is a collection of small and inexpensive embedded devices, with limited sensing, computing and communication capabilities, acting together to provide measurements of physical parameters or to identify events in known or unknown environments. Their real-life applications are rapidly emerging in a wide variety of domains, ranging from smart battlefields to natural habitat monitoring, precision agriculture, industrial process control or intelligent traffic management.

A large majority of the algorithms developed for WSNs are based on the assumption that all sensor nodes are aware of their position and, furthermore, of the position of nearby neighbor nodes. Since every measurement provided is strictly linked with the sensor node position in the field, a localization process with respect to a local/global coordinate system for each node must be carefully performed. Moreover, some other wireless sensor network related issues (e.g., geographic routing, sensing coverage estimation or nodes' sleep/wake-up procedures) might increase the need to accomplish nodes' localization by relying on location information.

The aim of localization is to supply the physical coordinates for all sensor nodes [1,2]. In the case of manually deployed WSNs, this localization process is almost straightforward. For random deployments in hostile terrain or dangerous battlefields often done through aerial scattering procedures from airplanes, guided missiles or balloons, the nodes' localization problem becomes complicated relying on special nodes that can detect their location automatically. These particular nodes are known as anchor or beacon nodes [3–8], being the cornerstones of every localization technique within global coordinates.

In order to identify their exact location, an almost general solution is to equip the beacon nodes with a Global Position System (GPS) receiver. This approach, even though it is based on a mature technology, has some drawbacks that make it impractical for many applications involving random deployments: (a) the GPS receivers are relatively expensive, energy demanding and bulky; (b) the GPS receivers may be confused by environmental obstacles, tall buildings, dense foliage, *etc.* [9–11]; (c) the GPS receivers cannot work when an insufficient number of satellites are directly visible [9,10,12]; and (d) the GPS receivers cannot solve completely the localization problem in the case of WSNs based on non-isotropic sensors (e.g., video-based sensor networks or multimedia-based sensor networks) because they cannot provide the orientation of the sensor camera.

In this paper we propose a new method to obtain the anchor node location that uses a digital compass (magnetometer), an image taken by a video camera and the exact location data for some geographically-located referential objects (e.g., solitary trees, electricity transmission towers, furnace chimneys, *etc.*) situated in the deployment area. This method, due to the low price of digital compasses, is particularly suitable for video- or multimedia-based wireless sensor networks [13–17] where the nodes already equipped with digital compasses (due to the necessity to estimate the cameras' fields of view) may simply become anchor nodes or anytime the GPS receiver is not considered to be an appropriate solution.

The remainder of the paper is organized as follows: Section 2 is a brief overview of anchor localization techniques. In Section 3 we describe the three-step methodology underlining the types of information needed. Section 4 presents the kernel of our approach—the triangulation-based procedure that fuses a valid photo image with the related compass information to obtain the beacon node position within global coordinates. In Section 5, an illustrative case study is depicted, while the conclusions are drawn in the last section.

## 2. Related Work

Due to cost and power consumption reasons, every node of a randomly deployed WSN cannot be equipped with localization components, only for a small number of them, named anchors. The anchor node localization procedure is basically done through triangulation or trilateration based on a number of known locations. The classical solution used in practice involves GPS receivers that use satellites to obtain the anchors' positions, but this approach is not generally viable due to some known shortcomings of GPS [18].

Reference [3] proposes a GPS-free approach designed for outdoor environments where a group of landmarks with known locations, can interact with the wireless nodes through radio signals. The method extends basic connectivity notion by adding an ideal theoretical model of radio behavior. Unfortunately, the solution cannot be applied in randomly deployed WSNs in harsh environments, where the grid infrastructure of pre-localized landmarks to support positioning localization infrastructure could not be set.

Another solution that uses prior known positions for anchor nodes is presented in [2]. The authors developed a method that aims to calculate the best placement for anchors in order to minimize the localization error and to reduce the number of required anchors, but it still requires pre-calculated deployment.

The anchor localization solution described in this paper does not rely on GPS receivers and does not require manual pre-calculated node placement. Instead, it uses a video camera and a compass to estimate the anchor position based on images captured by the camera and the compass information. It is very cost-effective, especially in the case of video- or multimedia-based wireless sensor networks, where only a compass is needed as supplementary node equipment.

## 3. Methodology Description

**Figure 1.**
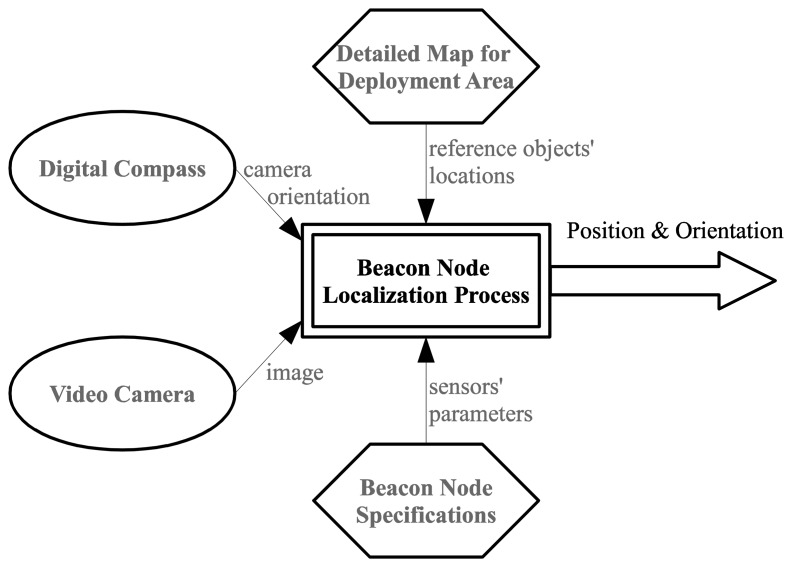
Information sources used in our beacon node localization method.

Anchor node localization is a key prerequisite for every localization technique in wireless sensor networks within global coordinates. Our method to determine the exact position of this special type of nodes is an alternative for using GPS devices in areas where the Earth's magnetic field is not disturbed by structures containing ferrous metals or by electronic interferences. It requires information captured by two sensors that equip the node (video and compass), exact positions of a few reference objects in the deployment area and some constructive parameters of the mentioned sensors, as follows (Figure 1):

- *A video image*—a valid snapshot provided by the anchor node's camera immediately after its random deployment in the field;
- *Compass information*—the orientation of the anchor node's camera provided by a digital compass module;
- *A set of reference objects' locations in the field of deployment*—we have to choose some referential objects (towers, lonely trees, electricity transmission towers, furnace chimneys, *etc.*) inside the area where the deployment is done and identify their precise locations using detailed maps (e.g., Google maps or military maps). To increase the efficiency of our proposed method, the reference objects have to be easily identifiable in the landscape when using automated object recognition algorithms [19,20];
- *Constructive parameters of the video camera and digital compass* (camera angle of view, camera depth of view, compass heading accuracy, *etc.*)—unlike the straightforward GPS-based localization, our approach involves some computations that require knowing the constructive parameters of sensors.

Based on information described above, a three-step methodology must be performed after the random deployment in the field:

(1) *The camera will take a valid photo* and send it to the base station (e.g., situated on the airplane which made the deployment) where enough computing and energy power is available;
(2) *Identify reference objects within photo*—this step is done using an object recognition procedure applied to the video image;
(3) *Obtain the exact location through a triangulation-based procedure*—the global coordinates of the anchor node are obtained by analyzing the video image in conjunction with the angle value provided by the compass using a triangulation technique. This procedure is presented in details in the following section.

## 4. Video/Compass Based Node Localization Using Triangulation

After anchor node deployment, a startup image is gathered by its video sensor and sent to the central point where an automatic shape recognition algorithm or even a human operator may identify objects from a given reference object set ℜ:

(1) $$ℜ=\left\{Obj_{i}|Obj_{i}\in \text{Obj}(\text{Map}),i\in I,i\in [1,N_{RO}]\right\}$$

This set is created prior to the sensor deployment and is based on analysis made upon known information about deployment field. It will contain *N_RO_* representative objects *Obj_i_* selected from the set *Obj*(*Map*) of all identifiable objects with known geographical positions from the deployment map. They have to be static (with fixed position) and easy to identify visually in images. They also have to be preferably thin in order to reduce the processing error. For objects localization we should use a military or a highly detailed topographic map if available, but even Google maps in conjunction with GPS localization offers a good estimation as demonstrated by the case study presented in Section 5.

**Figure 2.**
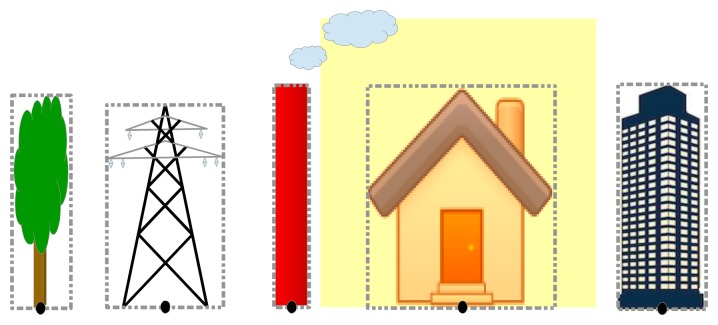
Examples of reference point selection for various objects.

All objects that belong to ℜ will be marked with a reference point calculated as the median point of the lower side of the bounding box framing the object. Position of the reference point is selected in order to be invariant to the height of the objects. However, if dimensions of some of the objects from ℜ are known, we can use this information to estimate the distance between the camera and these objects and therefore to validate or to make small correction after main localization algorithm is ended. Examples for choosing the reference point are depicted in Figure 2.

During setup processing, all reference objects from ℜ that could be identified in the setup images gathered from the anchors' sensors should be marked. Considering K deployed anchors, this procedure results in Γ*_k_* subsets of ℜ, one for each anchor node *k* ∈ [1…K]. A supplementary validation has to be applied on subsets in order to eliminate reference objects placed in positions inappropriate for localization procedure. This includes cases of obstructed objects, overlapping objects, and objects intersecting image edges. The further processing considers only the node set Σ containing anchors having *card*(Γ_k_) ≥ 2. To validate a candidate anchor the following algorithm is proposed, where σ*_i_* represents the image gathered in the setup phase from the anchor *i*:
1:**set** Γ*_i_ ≔* { }2:**foreach**
*o_j_* ∈ ℜ **do**3: **if** σ*_i_* *contains* *o_j_*
**then** Γ*_i_≔* Γ*_i_* ∪ {*o_j_*}4:**foreach**
*o_k_* ∈ Γ*_i_*
**do**5: **if** *obstructed or partially visible* (*o_k_*, σ*_i_*) **then**6:  Γ*_i_ ≔* Γ*_i_* / {*o_k_*}7:**foreach**
*o_m_* ∈ Γ*_i_*
**do**8: **foreach**
*o_n_* ∈ Γ*_i_*
**do**9:  **if** (m != n) ***and*** (*o_m_* ∩ *o_n_* != { }) **then**10:  Γ*_i_≔* Γ*_i_* / {*o_n_*, *o_m_*}11:**if *card***(Γ*_i_*) >= 2 **then**12: * validate ***anchor i***

The next step of localization algorithm is based on the well-known triangulation method [21,22]. Triangulation is the process of determining the location of a point by measuring angles to it from other two points whose position on a map is known. In case of more than two reference points, triangulation could be applied repetitively on all possible pairs formed by two distinctive points in order to decrease the error by averaging the results.

**Figure 3.**
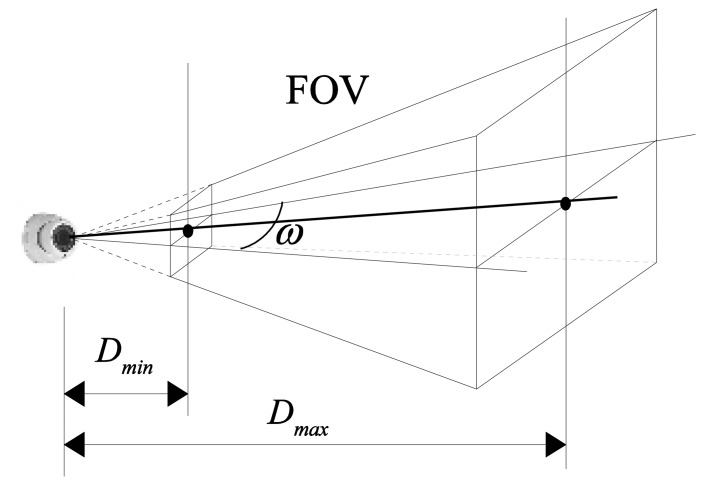
The camera model.

The first step consists in determining the angle of the vertices formed by the camera and the two reference points. For this we consider the camera model presented in Figure 3. The model is expressed by the parameters of its Field of View (*FOV*): the angle of view *ω*, the minimum (*D_min_*) and the maximum depth of field (*D_max_*). For an ideal camera, the field of view represents the volume of a 3D pyramid containing the part of the environment visible to the camera. The angle of view is considered on a horizontal plane projection of the *FOV*. All points from this volume are visible in the captured image as long as any obstacles do not obstruct the projection ray from the point to the optical center of the camera. However, the depth of field of a real camera is constrained due to limited resolution and distortion of its lenses. Some objects that are too close or too far from the lens will appear blurred. Therefore, the field of view depth will be limited in a range described by an [*D_min_*, *D_max_*] interval.

To determine the absolute camera position using two reference objects we define first a Cartesian reference system. Considering the map of the deployment area we choose and mark an arbitrary point situated near lower left corner of the area. Then we measure on the map the position of this point in geographic coordinates as a (latitude, longitude) pair. Next, we consider this point as the origin of a Cartesian referential system having the y-axis oriented along the North direction on the map, and the x-axis pointing toward East.

**Figure 4.**
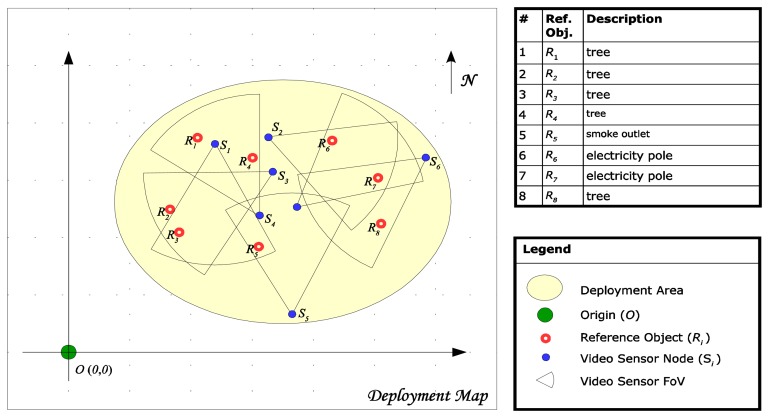
Reference system defined for network deployment area.

Figure 4 depicts an example of a referential system defined on a topographical map. For easy identification, the reference objects are marked with red circles on the map, while the chosen origin is highlighted in green.

Then we can express the absolute reference objects location into this referential system. For this, we use the haversine Equation (2) based on inverse tangent function to calculate the great circle distance between two points on the Earth's surface [23]:

(2) $$\left\{ \begin{array}{l} a=\sin^{2}\left(\frac{\Delta \varphi }{2}\right)+\cos\left(\varphi_{1}\right)\cos\left(\varphi_{2}\right)\sin^{2}\left(\frac{\Delta \lambda }{2}\right)\\ c=2\cdot \arctan\left(\frac{\sqrt{a}}{\sqrt{1-a}}\right)\\ d=c\cdot R \end{array}\right.$$

where (*φ_1_*, *λ_1_*) and (*φ_2_*, *λ_2_*) represent the latitude and the longitude of the two points; Δ*φ* and Δ*λ* are the difference of latitude and longitude; and *R* is an approximation of the radius of the average circumference of the Earth with a value of 6,372.8 km. The value of *a* represents the square of half the chord length between the points and *c* represents the angular distance in radians.

Therefore, to calculate the absolute coordinates of the reference objects in the proposed Cartesian reference system, we can consider the distances between the origin point and the projection of the corresponding objects reference points on the two axes. These projections will have Δ*φ* = 0 on the x-axis and Δ*λ* = *0* on the y-axis. Considering (*φ_o_*, *λ_o_*) and (*φ_RP1_*, *λ_RP1_*) as the geographical coordinates of the origin and the coordinates of the first reference point, we can use Equations (3) and (4) for absolute position computation:

(3) $$\left\{ \begin{array}{l} a^{\prime }=\cos^{2}\left(\varphi_{RP1}\right)\sin^{2}\left(\frac{\Delta \lambda }{2}\right)\\ c^{\prime }=2\cdot \arctan\left(\frac{\sqrt{a^{\prime }}}{\sqrt{1-a^{\prime }}}\right)\\ x_{RP1}=c^{\prime }\cdot R \end{array}\right.$$

(4) $$\left\{ \begin{array}{l} a^{\prime \prime }=\sin^{2}\left(\frac{\Delta \varphi }{2}\right)\\ c^{\prime \prime }=2\cdot \arctan\left(\frac{\sqrt{a^{\prime \prime }}}{\sqrt{1-a^{\prime \prime }}}\right)\\ y_{RP1}=c^{\prime \prime }\cdot R \end{array}\right.$$

Then, the position of the second reference point (*x_RP2_*, *y_RP2_*) will be computed using a similar set of formulas.

**Figure 5.**
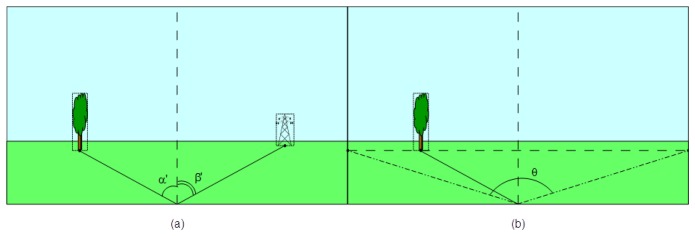
Computation of reference points bearing.

To finalize the first step we use the image captured by the sensor camera where the two reference objects were identified. The aim is to determine the angles α′ and β′ formed by the camera axis and the two reference points. A vertical line drawn on the center of image and therefore aligned with the camera's direction of sight is used as reference. Angles are measured counterclockwise relative to this direction, as depicted in Figure 5a. However, these values are hardly influenced by camera's tilt, perspective and angle of view. To eliminate this influence we have to consider the ratio between the angle of view *ω* and the measured angle of view *θ* corresponding to reference point depth. The measured angle of view is obtained from the triangle formed in image by the center of the bottom margin and the two vertices that corresponds to reference point perspective. These vertices are determined by intersection of a horizontal line traversing the reference point with the left and right margins as presented in Figure 5b.

Starting from these values the next step consists in computing the values of the reference angles relative to the geographic North. First we use the compass measurement to obtain the orientation of the camera axis denoted here by the angle γ. In addition we should consider the magnetic declination of the geographical position of the deployment field. Magnetic declination represents the angle between geographic and magnetic North at a specific location and can be positive or negative [24]. Despite the relative vicinity of the geomagnetic North Pole and the geographic North Pole the error could be significant. The correction consists in adding this magnetic declination *mag_decl* to the measured angle. The final values of reference points' angles α and β are given by the Equation (5):

(5) $$\{\begin{array}{c} \alpha =\gamma +\alpha^{\prime }\cdot \left(\omega /\theta_{RP_{1}}\right)+\text{mag}\_\text{decl}\\ \beta =\gamma +\beta^{\prime }\cdot \left(\omega /\theta_{RP_{2}}\right)+\text{mag}\_\text{decl} \end{array}$$

**Figure 6.**
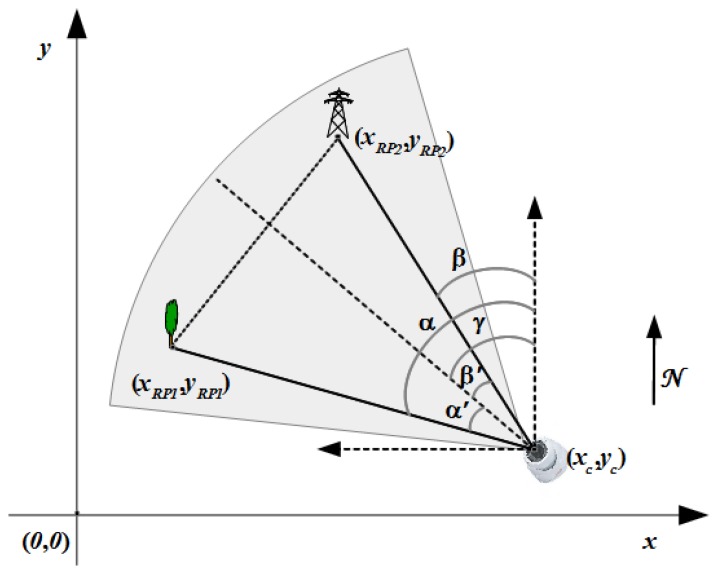
Position estimation using triangulation.

The last step is the triangulation process itself, presented in Figure 6. It implies the computation of the position (*x_c_*,*y_c_*) of the sensor camera on the Cartesian reference system defined on the map. For this we use the following equations:

(6) $$\{\begin{array}{c} \left(y_{c}-y_{RP_{1}})=tg(\pi /2-\alpha )(x_{c}-x_{RP_{1}}\right)\\ \left(y_{c}-y_{RP_{2}})=tg(\pi /2-\beta )(x_{c}-x_{RP_{2}}\right) \end{array}$$

Therefore, the position is calculated as:

(7) $$x_{c}=\frac{\left(y_{RP_{2}}-y_{RP_{1}})+(x_{RP_{1}}tg(\pi /2-\alpha )-x_{RP_{2}}tg(\pi /2-\beta )\right)}{tg(\pi /2-\alpha )-tg(\pi /2-\beta )}$$

(8) $$y_{c}=(x_{c}-x_{RP_{2}})tg(\pi /2-\beta )+y_{RP_{2}}$$

The final result of entire localization process is expressed by the triplet {*x_c_*, *y_c_*, *γ*}. Together with existing FOV information {*ω*, *D_min_*, *D_max_*}, it allows implementation of complex application based on localized anchor node. The accuracy of proposed localization algorithm is influenced by several factors:

- The approximation of the radius of the average Earth circumference, which results from the mean of radius variation. However, this variation from the average radius to meridian (6,367.45 km) or equator (6,378.14 km) is less than 0.08%.
- The precision of localization of reference objects. If we use a GPS for estimation we can rely on a precision around 10 m, depending of the number and position of available satellites [25].
- The precision of digital compass measurement in general is less than 1.0 degree. For example the three-axis HMR3000 Digital Compass Module (Honeywell, Plymouth, MN, USA) provides 0.5° accuracy [26].
- The precision of reference points' angles estimation depends on the method used to identify reference objects into setup images. However, by favoring very thin objects during creation of ℜ we can hardly increase the accuracy.

## 5. Case Study

**Figure 7.**
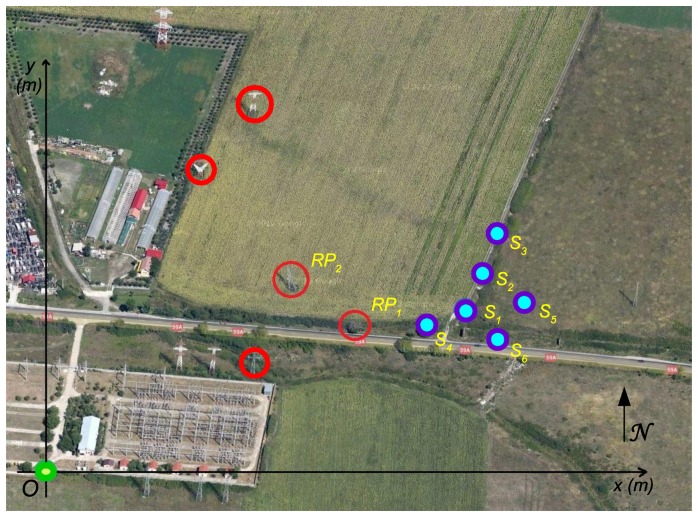
Network deployment area with highlighted reference objects on a Google map.

To demonstrate the effectiveness of the presented algorithm we consider six anchor nodes, deployed in an area near km-2 of route RO DN59A. The deployment configuration is presented in Figure 7 using a Google map [27], where the reference objects are marked with red circles while the sensors *S_i_* are marked in blue.

The chosen origin *O* is highlighted in green and has the geographical coordinates of 45.759941° North latitude and 21.158411° East longitude. The magnetic declination in this area is 4.39984°. All anchor nodes are equipped with VGA sensors characterized by a measured angle of view *ω* = 39° and an approximate FOV depth in the range [0.6 m, 450 m].

**Figure 8.**
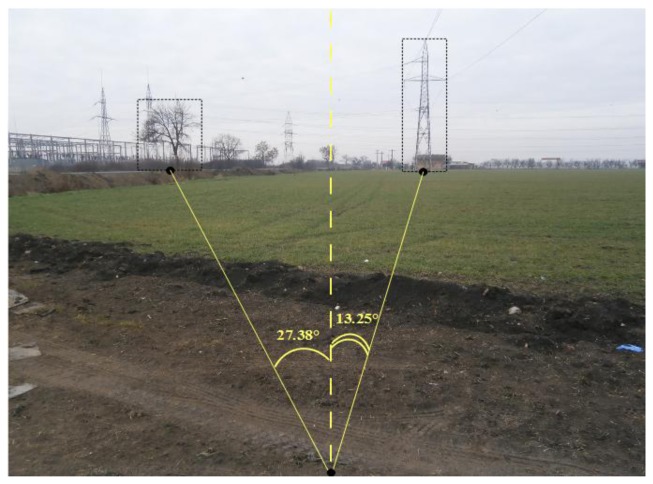
Reference points angles measurement for S_1_.

In the following, we present in detail the localization steps for the first node, the results for all nodes being summarized in Table 1. In the image taken by the *S_1_* node (Figure 8), a tree and an electricity pole, used further as reference objects *RP_1_* and *RP_2_*, had been identified.

For *S_1_*, the bearing *γ*, measured by the digital compass, was 282.2° WNW. The reference points' angles were measured as presented in Figure 8 and have the values α′ = −27.38° and β′ = 13.25°. The angles of view *θ_RP1_* and *θ_RP2_* corresponding to the depth of the reference points are 92.39° and 92.19°, respectively. The correction factors *(ω/θ)* are in this case 2.3689 and 2.3638. Using Equation (4) we computed the corrected reference points' angles as α = 275.0471° and β = 292.2051°.

Using Equations (6) and (7) we obtained the localization coordinates in local referential system:

$$\begin{array}{l} x_{c}=276.55\text{m}\\ y_{c}=131.65\text{m} \end{array}$$

In order to estimate the error we used a supplementary GPS measurement of sensor *S_1_* position and, after applying Equations (2) and (3), we obtained:

$$\begin{array}{l} x_{\text{GPS}}=277.18\text{m}\\ y_{\text{GPS}}=139.14\text{m} \end{array}$$

Table 1 summarizes the results obtained after repeating this procedure for all six nodes deployed. It presents the measured and estimated positions for all nodes and the absolute position errors, relative to GPS provided location, defined as:

(9) $$\Delta d=\sqrt{{\left(x_{\text{GPS}}-x_{C}\right)}^{2}+{\left(y_{\text{GPS}}-y_{C}\right)}^{2}}$$

where *x_GPS_* and *y_GPS_* represent the coordinates in the defined Cartesian system calculated using GPS measurements and *x_C_* and *y_C_* the estimated coordinates using compass and image information.

As resulting from Table 1, in our experiment the maximum absolute position error is around 11 m. The mean of absolute position error for all six nodes is 7.12 m, with a standard deviation of 2.36 m. Moreover, ignoring the compass error and considering the GPS positioning error around 10 m [25] we can reasonable estimate a maximum localization error for the mentioned experimental deployment at a value less than 25 m using presented method.

## 6. Conclusions

Localization is a crucial procedure for random deployed wireless sensor networks. It is generally based on anchor nodes with efficient capabilities to automatically acquire their position in global coordinates. In this paper, we proposed a new anchor node localization technique meant for special cases where GPS receivers are unavailable or too expensive. Using a triangulation technique based on a video image and compass information our method displays a reasonable precision. Moreover, our method is especially appropriate for non-isotropic sensor networks, where magnetometers providing the orientation of the sensor cones are mandatory.

## Figures and Tables

**Table 1.** Overall localization results for all six deployed nodes.

**Sensor #**	***x****_GPS_* **(m)**	***y****_GPS_* **(m)**	***x****_C_* **(m)**	***y****_C_* **(m)**	**Absolute Position Error Δ*d* (m)**
1	277.18	139.14	276.55	131.65	7.52
2	296.43	189.31	298.63	192.47	3.85
3	310.16	238.80	299.87	234.54	11.14
4	258.95	131.58	261.49	137.25	6.21
5	336.31	156.49	332.28	152.99	5.34
6	319.71	124.90	312.85	119.61	8.66

## Notes

**Authors Contributions** The authors contributed equally to this work.

## References

[1] Bachrach J., Taylor C. (2005). Localization in Sensor Networks. Handbook of Sensor Networks: Algorithms and Architectures.

[2] Kunz T., Tatham B. (2012). Localization in wireless sensor networks and anchor placement. J. Sens. Actuator Netw..

[3] Bulusu N., Heidemann J., Estrin D. (2000). GPS-less low-cost outdoor localization for very small devices. IEEE Pers. Commun. Mag..

[4] Cota-Ruiz J., Rosiles J.-G., Sifuentes E., Rivas-Perea P. (2012). A low-complexity geometric bilateration method for localization in wireless sensor networks and its comparison with least-squares methods. Sensors.

[5] Paul A.K., Sato T. (2013). Detour path angular information based range-free localization in wireless sensor network. J. Sens. Actuator Netw..

[6] Sahoo P.K., Hwang I. (2011). Collaborative localization algorithms for wireless sensor networks with reduced localization error. Sensors.

[7] Chen X., Chen J., He J., Chen C. (2012). The selection of reference anchor nodes and benchmark anchor node in the localization algorithm of wireless sensor network. Intell. Autom. Soft Comput..

[8] Kim T., Shon M., Kim M., Kim D.S., Choo H. (2012). Anchor-node-based distributed localization with error correction in wireless sensor networks. Int. J. Distrib. Sens. Netw..

[9] Williams B. (2008). Intelligent Transport Systems Standardization.

[10] Feiner S., MacIntyre B., Höllerer T., Webster A. (1997). A touring machine: Prototyping 3D mobile augmented reality systems for exploring the urban environment. Pers. Technol..

[11] Vecchio M., López-Valcarce R., Marcelloni F. (2012). A two-objective evolutionary approach based on topological constraints for node localization in wireless sensor networks. Appl. Soft Comput..

[12] Rodríguez A., Bergasa L.M., Alcantarilla P.F., Yebes J., Cela A. Obstacle Avoidance System for Assisting Visually Impaired People.

[13] Costa D.G., Guedes L.A., Vasques F., Portugal P. (2013). Energy-efficient packet relaying in wireless image sensor networks exploiting the sensing relevancies of source nodes and DWT coding. J. Sens. Actuator Netw..

[14] Costa D.G., Guedes L.A. (2010). The coverage problem in video-based wireless sensor networks: A survey. Sensors.

[15] Akyildiz I.F., Melodia T., Chowdury K.R. (2007). Wireless multimedia sensor networks: A survey. IEEE Wirel. Commun..

[16] Pescaru D., Istin C., Naghiu F., Gavrilescu M., Curiac D. Scalable Metric for Coverage Evaluation in Video-Based Wireless Sensor Networks.

[17] Farooq M.O., Kunz T. (2014). Wireless sensor networks testbeds and state-of-the-art multimedia sensor nodes. Appl. Math. Inf. Sci..

[18] Rizos C. Trends in Geopositioning for LBS, Navigation and Mapping.

[19] Bennamoun M., Mamic G.J. (2002). Object Recognition. Fundamentals and Case Studies. Advances in Computer Vision and Pattern Recognition.

[20] Campbell R.J., Flynn P.J. (2001). A survey of free-form object representation and recognition techniques. Comput. Vis. Image Underst..

[21] Zhou H., Wu H., Xia S., Jin M., Ding N. A Distributed Triangulation Algorithm for Wireless Sensor Networks on 2D and 3D Surface.

[22] Bal M., Liu M., Shen W., Ghenniwa H. Localization in Cooperative Wireless Sensor Networks: A Review.

[23] Sinnott R.W. (1984). Virtues of the haversine. Sky Telesc..

[24] Easa S. (1989). Analytical solution of magnetic declination problem. J. Surv. Eng..

[25] Hofmann-Wellenhof B., Lichtenegger H., Collins J. (2001). Global Positioning System: Theory and Practice.

[26] Honeywell International Inc. Honeywell Magnetic Sensors—HMR3000. http://www.magneticsensors.com/magnetometers-compasses.php.

[27] Google Google Maps. https://maps.google.com/.

